# Hierarchical Nonlocal Residual Networks for Image Quality Assessment of Pediatric Diffusion MRI with Limited and Noisy Annotations

**DOI:** 10.1109/TMI.2020.3002708

**Published:** 2020-10-28

**Authors:** Siyuan Liu, Kim-Han Thung, Weili Lin, Dinggang Shen, Pew-Thian Yap

**Affiliations:** Department of Radiology and Biomedical Research Imaging Center (BRIC), University of North Carolina at Chapel Hill, NC, U.S.A.; Department of Radiology and Biomedical Research Imaging Center (BRIC), University of North Carolina at Chapel Hill, NC, U.S.A.; Department of Radiology and Biomedical Research Imaging Center (BRIC), University of North Carolina at Chapel Hill, NC, U.S.A.; Department of Radiology and Biomedical Research Imaging Center (BRIC), University of North Carolina at Chapel Hill, NC, U.S.A.; Department of Brain and Cognitive Engineering, Korea University, Seoul, Korea.; Department of Radiology and Biomedical Research Imaging Center (BRIC), University of North Carolina at Chapel Hill, NC, U.S.A.

**Keywords:** Image quality assessment, hierarchical nonlocal residual networks, semi-supervised learning, self-training

## Abstract

Fast and automated image quality assessment (IQA) for diffusion MR images is a crucial step for swiftly making a rescan decision during or after the scanning session. However, learning a model for this task is challenging as the number of annotated data is limited and the annotation labels might not always be correct. As a remedy, we will introduce in this paper an automatic image quality assessment (IQA) method based on hierarchical non-local residual networks for pediatric diffusion MR images. Our IQA is performed in three sequential stages, i.e., 1) *slice-wise IQA*, where a nonlocal residual network is first pre-trained to annotate each slice with an initial quality rating (i.e., pass/questionable/fail), which is subsequently refined via iterative semi-supervised learning and slice self-training; 2) *volume-wise IQA*, which agglomerates the features extracted from slices of a volume, and uses a nonlocal network to annotate the quality rating for each volume via iterative volume self-training; and 3) *subject-wise IQA*, which ensembles the volumetric IQA results to determine the overall image quality pertaining to a subject. Experimental results demonstrate that our method, trained using only samples of modest size, exhibits great generalizability, and is capable of conducting rapid hierarchical IQA with near-perfect accuracy.

## I. Introduction

Diffusion magnetic resonance imaging (dMRI) is a powerful tool for probing tissue microstructure and investigating white matter pathways [1–3]. However, it is susceptible to imaging artifacts caused by motion, which is especially common for pediatric subjects. Artifacts could bias analyses, invalidate conclusions, and weaken interpretability [4, 5]. Therefore, extra care must be taken in ensuring data quality prior to subsequent processing and analysis.

Image quality assessment (IQA) is key in determining whether an acquired image is usable and whether a re-scan is necessary. Further decisions can be made based on IQA to exclude affected slices, volumes, and subjects or to correct for artifacts. IQA can be carried out either subjectively via visual inspection by a human rater, or objectively by a computer algorithm [6, 7]. Subjective IQA can be time-consuming, labor-intensive, costly, error-prone, and inconsistent [8]. IQA is especially challenging in dMRI as typically a series of volumes are acquired for each subject. In time-sensitive studies involving for example newborns, scheduling rescans is often not an option since the brain changes literally on a daily basis. Therefore, a fast, reliable, accurate, and fully-automated objective IQA for dMRI is highly desirable.

Generally, objective IQA can be grouped into three categories based on the availability of reference images, i.e., 1) full-reference IQA (FR-IQA), which requires a pristine image as reference; 2) reduced-reference IQA (RR-IQA), which requires partial information from a reference image; and 3) no-reference IQA (NR-IQA), which requires no information from the reference image. Specifically, FR-IQA methods measure the quality of an image by comparing it with a reference using evaluation metrics [9–12]. RR-IQA methods utilize only a limited number of features extracted from a reference [13] to provide near FR-IQA performance. However, FR-IQA and RR-IQA have limited practical applications as full or partial information from a pristine reference image is not always available. In contrast, NR-IQA methods [14–17] do not require any information from a reference image and is well-suited for practical applications. Currently, most NR-IQA methods are designed for natural 2D images, while diffusion MR images are 3D with intensity distributions and artifacts that differ greatly from natural images.

Recently, deep neural networks (DNNs), particularly convolutional neural networks (CNNs), have been demonstrated to have great potential for IQA [18]. Instead of hand-crafting features always developed in-house [19], CNNs automatically learn image features that are pertinent to IQA. However, the performance of these deep learning methods generally depend on the number of labeled training samples and the correctness of their corresponding labels. Medical images, particularly diffusion MR images, are typically limited on the number of annotated images, as annotating them requires a huge amount of efforts from experts. For example, it is labor-intensive and time-consuming to annotate quality scores for all the image volumes in dMRI of all the subjects to train a volume-wise IQA network. To address the issue of limited annotated data, Graham et al. [20] proposed to use simulated images to augment training data. However, simulated images may not fully capture the characteristics of real acquired images. Another less labor-insensitive alternative is to annotate each volume (or scan session) with a single quality label. This is, however, inaccurate and will introduce label noise as each volume (or scan session) might inevitably contain a mixture of good or bad image slices (or volumes). Furthermore, the label noises might also be introduced by 1) mislabeling or unreliable labeling due to human error; and 2) inconsistent labeling among different experts.

In this paper, on the basis of our preliminary version [21], we address the above issues by proposing a deep learning based hierarchical IQA method that requires only a small amount of annotated volumes for training and is robust to annotation errors. To the best of our knowledge, this is the first deep learning based dMRI IQA method that caters to limited annotated images and label noise. The key features of our method are summarized as follows:

1. Our method consists of three sequential stages: slice-wise IQA using a slice-wise QA network (SQA-Net), volume-wise IQA by agglomerating the extracted slice features using a volume-wise QA network (VQA-Net), and subject-wise IQA by ensembling the volume-wise IQA results using a decision rule. Unlike previous deep learning based dMRI IQA methods that only provide volume assessment, our method simultaneously provides assessment results at different levels, i.e., slice, volume and subject.
2. For better computational efficiency and feature expression capability, we construct SQA-Net by incorporating depthwise separable convolutions (DSConv) and nonlocal mean operation into residual blocks. Similarly, VQA-Net consists of a nonlocal block and a classifier block. The computation-reduction property of DSConv and the information fusion property of nonlocal block enable SQA-Net and VQA-Net to be trained efficiently using only a small number of data, making the networks suitable for real-time IQA.
3. We utilize semi-supervised learning to deal with the scenario where we have a small amount of labeled data but a large amount of unlabeled data. Specifically, using a small number of labeled samples, we pre-train SQA-Net, and then use it to select confident slices from unlabeled slices, which are then used together with the labeled slices to re-train SQA-Net. This labeling and re-training process is iterated until the annotation of unlabeled slices stabilizes.
4. We employ an iterative self-training mechanism to remove or relabel unreliable labels to improve assessment accuracy of SQA-Net and VQA-Net.

The rest of this paper is organized as follows. In Section II, we briefly review related work on learning with limited labeled data affected by label noise. Section III describes the architectures of slice-/volume-wise quality assessment networks, semi-supervised learning, and slice/volume self-training. We report the experimental results in Section IV and conclude in Section VI.

## II. Related Work

### A. Learning with Limited Labeled Data

When only a small quantity of labeled data are available, the parameters of CNNs may not be learned properly. This problem can be mitigated by either 1) data augmentation, 2) pre-training the network using an alternative but related large dataset and then fine-tuning it using the original small dataset, or 3) label the unlabeled data.

Data augmentation is a popular approach to significantly increase the number of samples to train an effective deep learning network. This strategy has been used for dMRI IQA in [20] with data simulated using noise and motion artifacts. However, the trained model is only sensitive to the simulated artifacts, and cannot detect other types of artifacts in dMRI data.

Transfer learning methods adopt the second idea [22, 23]. Network weights are first initialized by pre-training the network using a large related dataset, and are then fine-tuned using a relatively smaller dataset related to the target task. However, the effectiveness of transfer learning might be deteriorated when the two datasets are only loosely related. This is especially true for dMRI IQA, where a larger alternative dataset with matching characteristics is seldom available.

Semi-supervised learning [24] adopt the third idea and trains networks/models with a small amount of labeled data and a large amount of unlabeled data. Strategies to label unlabeled data can be divided into three classes: generative methods [25], graph methods [26, 27], pseudo-labeling methods [28, 29]. *Generative methods* assume a label-parameterized model for labeled or unlabeled data. However, performance degrades with model inaccuracy. *Graph methods* represent each labeled or unlabeled data sample as a node in a graph and define the edge between two nodes as sample similarity computed with a Gaussian kernel. Fixing the labels of labeled data, the labels of unlabeled data are iteratively updated until convergence. However, graph methods have large storage requirements and the graph has to be reconstructed and updated for new data. *Pseudo-labeling methods* assign initial pseudo-labels to unlabeled data via an initial model that is trained using a small-scale labeled dataset. Then, the pseudo-labels of the unlabeled data are updated via an optimized model that is obtained by retraining the initial model using both labeled and pseudo-labeled datasets.

### B. Learning with Noisy Labels

Noisy labels can result in performance deterioration and increase in model complexity [30]. Machine learning methods proposed to address this issue can be grouped into two categories: direct and indirect learning with noisy labels.

#### Direct learning with noisy labels:

Some researches proposed to use reweighted or rectified loss functions that are robust to label noises [31–33], such as ramp loss [34] and unhinged loss [35]. However, it is shown in [36] that these loss functions are not completely robust to label noise.

An alternative is to utilize classifier algorithms that are robust to label noise, such as SVM, and random forest. Nevertheless, the robustness of these methods is limited, as shown empirically in [37]. Label noise models can also be embedded into the classification model to alleviate the influence of label noise [38–43]. However, this approach is largely dependent on the availability and quality of prior label noise information. Furthermore, this approach increases the complexity of learning algorithms and may result in overfitting due to the additional model parameters.

#### Indirect learning with noisy labels:

Some label noise cleansing methods are proposed to remove or relabel problematic samples before training [44, 45]. However, the removal operation may lead to overcleansing in data-imbalance cases [46]. That is, minority classes might be entirely removed, causing degradation in classification performance. In comparison, relabeling mislabeled data maintains the sample size, but incorrect relabeling may also lead to performance degradation. In this context, our method adopts the label noise cleansing method to simultaneously maintain the sample size and remove unreliable data.

## III. Method Architecture

**Fig. 1.**
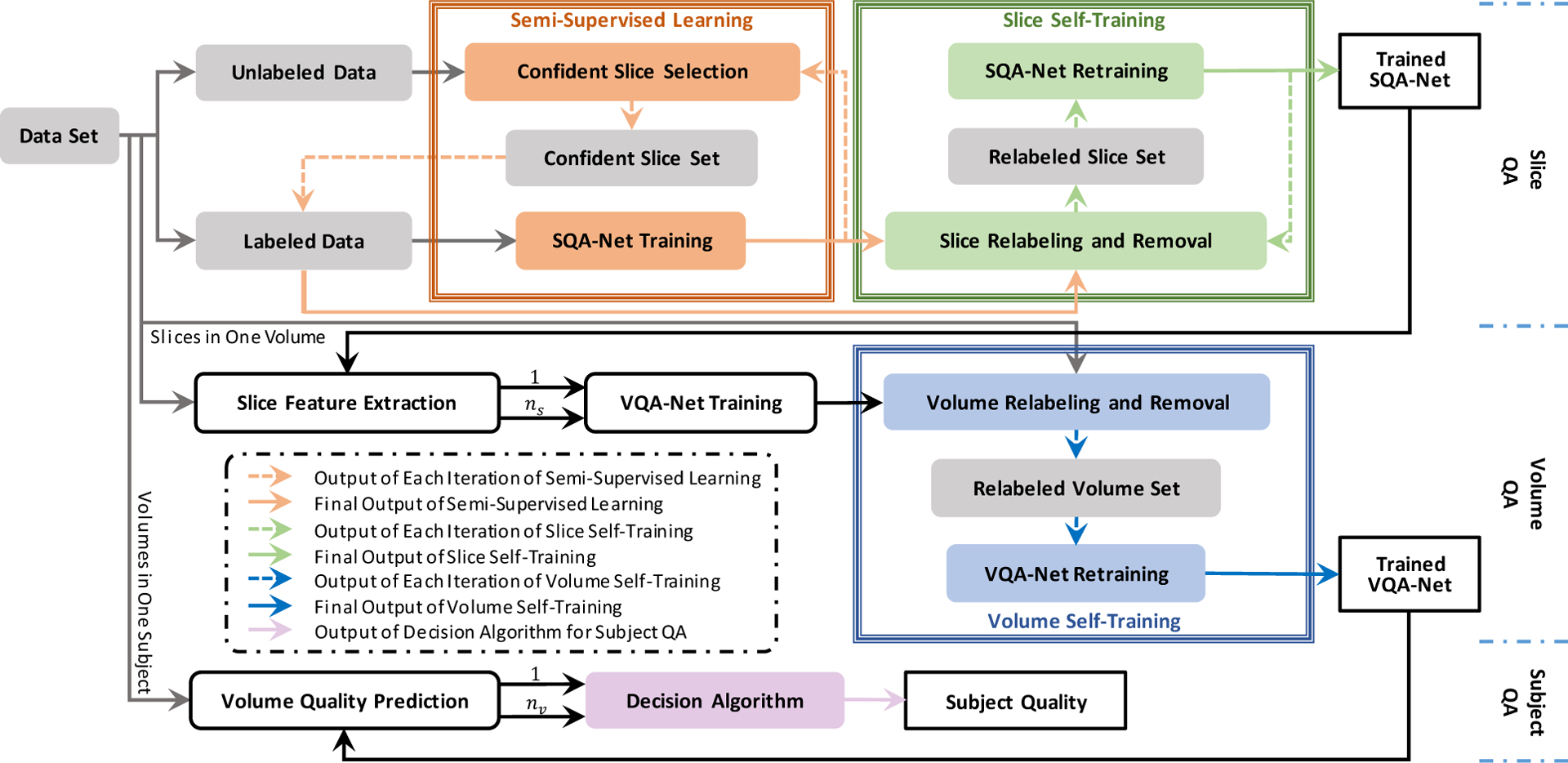
Overview of our IQA method, which includes slice IQA, Volume IQA, and subject IQA. *n*_*s*_ and *n*_*v*_ denote the number of slices in one volume and the number of volumes in one subject, respectively.

Fig. 1 shows an overview of our dMRI IQA method, which consists of three stages: slice-wise, volume-wise, and subject-wise IQA stages. The slice-wise IQA stage is designed to predict the quality rating of each slice, which involves two iterative processes, i.e., semi-supervised learning and slice self-training. The volume-wise IQA stage is introduced to evaluate the quality of each volume based on the quality of the constituent slices. Volume-wise IQA is iterative refined via a self-training algorithm. The subject-wise IQA stage agglomerates the quality ratings of volumes belonging to this subject. The details on each component of our dMRI IQA method will be described next.

### A. Slice Quality Assessment Network (SQA-Net)

**Fig. 2.**
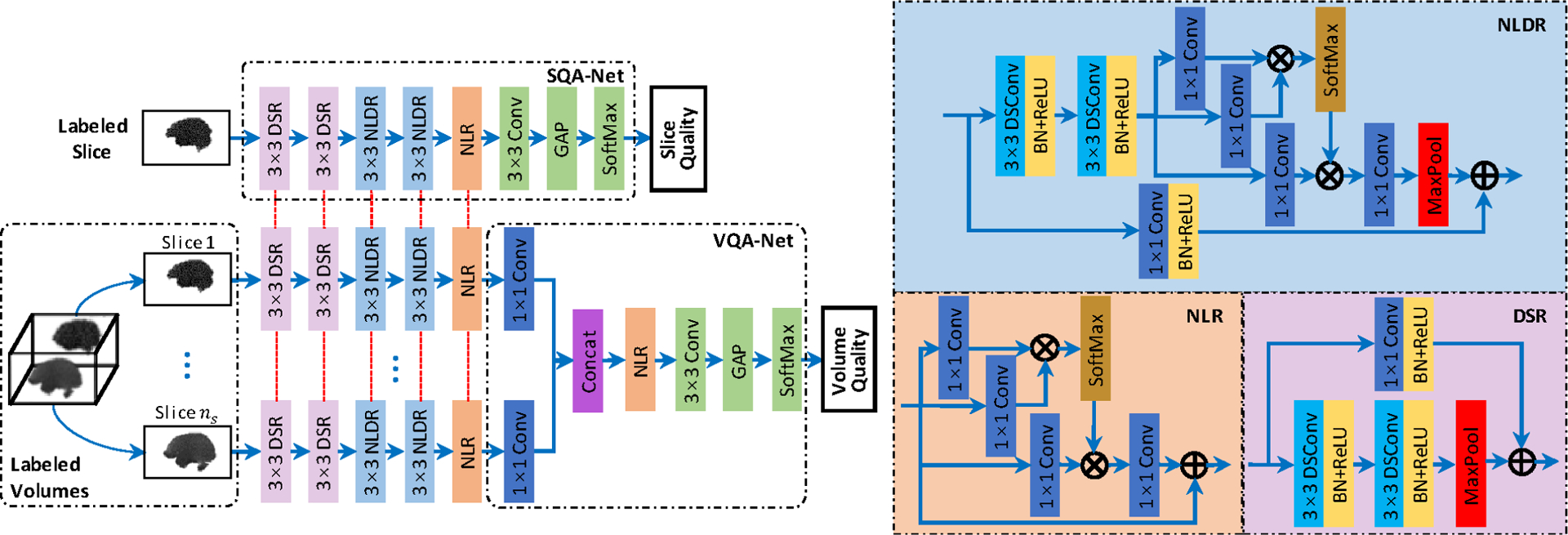
Architecture of our slice and volume assessment networks, i.e., SQA-Net and VQA-Net. ⊗: matrix multiplication, ⊕: element-wise summation. Red dashed line denotes weight sharing. *n*: the number of slices included in one volume.

Our slice quality assessment network (SQA-Net) is designed with both accuracy and speed in mind. Fig. 2 shows the proposed network, consisting of four types of network blocks, i.e., depthwise separable residual (DSR) block (in light pink), nonlocal depthwise separable residual (NLDR) block (in light blue color), nonlocal residual (NLR) block (in orange), and classifier (CLF) block (in green). The DSR blocks and NLDR blocks extract low- and high-level features, respectively, whereas the NLR block [47] computes the response function at each position as a weighted summation of all features in the feature maps. The CLF block outputs three probability values indicating whether a slice is “pass”, “questionable”, or “fail”. The number of channels for each network block from the first DSR block to CLF block is 32, 64, 28, 256, 256, and 3, respectively.

Our IQA framework is holistic in that it relies on both local and global information. That is, it considers relationship not only among local features, but also among non-local features. While a typical convolutional layer can extract local information easily, we need multiple layers of convolution operations to extract non-local information. This is inefficient, computationally expensive, and unable to directly capture dependencies. Thus, most of our network blocks adopt two major network architectures, i.e., the non-local network architecture, where the response function at each position is computed as the weighted summation of features at both short-range (local) and long-range (global) positions, and the residual network architecture, which is devised to avoid the vanishing/exploding gradient issue caused by the increase of network layers and connections. In this way, our network can assess the quality of an image holistically to output a more accurate rating.

The DSR block is constructed by integrating 3×3 depthwise separable convolutions (DSConv) layers [48] with unit stride into a residual block, where each DSConv layer is followed by batch normalization (BN) and rectified linear unit (ReLU) activations, and a 2×2 max-pooling layer is adopted to down-sample the intermediate features. The shortcut branch of DSR block is implemented by a 1×1 standard convolution with a 2*×*2 stride.

The NLDR block is based on DSR block, where a nonlocal network block is added before max-pooling layer. The non-local network block computes the output at the *i*-th location $r_{i}\in {\mathbb{R}}^{c}$ of the nonlocal network block as the weighted sum of all input features for a *c*-channel *h* × *w* input feature map *x*, given as

(1) $$r_{i}=\frac{1}{C_{i}(x)}\sum_{\forall j}f(x_{i},x_{j})g(x_{j}),$$

where the weight function *f*(·) computes the pairwise relationship (e.g., similarity) between feature vectors at two locations, *g*(·) computes a representation of the input feature, and *C*_*i*_(*x*) = Σ_∀*j*_ *f*(*x*_*i*_, *x*_*j*_) is a normalization factor. In this work, the weight function *f*(*x*_*i*_, *x*_*j*_) is defined in the following embedded Gaussian form:

(2) $$f(x_{i},x_{j})={\text{exp}}^{\phi^{T}\left(x_{i}\right)\psi \left(x_{j}\right)},$$

where *ϕ*(·) and *ψ*(·) are the unary kernel functions that are implemented with 1×1 convolution kernels with unit stride, thus making $\frac{1}{C_{i}(x)}f(x_{i},x_{j})$ a softmax function in implementation.

The NLR block is a combination of nonlocal and residual network architectures. The CLF block is realized with a 3×3 convolution layer with unit stride, global average pooling (GAP), and softmax activation function.

### B. Volume Quality Assessment Network (VQA-Net)

Using the output of the NLR block in SQA-Net (i.e., some kind of high-level features) for slices belonging to the same volume as input, we design a volume quality assessment network (VQA-Net) to determine the quality for this volume. Specifically, we first employ a 1×1 convolution to reduce the channel dimension of the feature representation for each slice from 256 to 16. Then, using the concatenated dimension-reduced features as inputs, we subsequently employ a NLR block and a CLF block to output the quality label (i.e., “pass”, “questionable” or “fail”) for the current volume based on the quality rating with the highest probability.

### C. Semi-Supervised Learning in SQA-Net

As mentioned previously, we have limited number of labeled samples. Thus, we employ semi-supervised learning when training SQA-Net (orange box in Fig. 1) to make full use of plentiful unlabeled data by progressively selecting confident slices from the unlabeled dataset to be included in labeled dataset for retraining the SQA-Net.

Specifically, we first utilize SQA-Net that has been pre-trained with the labeled dataset to predict the quality label for each slice of unlabeled volumes. We then pseudo-label each slice with the predicted quality label. Slices with high confident pseudo-labels (maximal probability greater than a predefined threshold, i.e., 0.9) are then merged with the labeled dataset to retrain SQA-Net. This is done iteratively until the network converges, i.e., when performance improvement is minimal.

### D. Slice Self-Training

We only have volumetric label and assuming the label for each slice in a volume to be the same, which is inaccurate. To deal with inaccurate slice labels, we propose a slice self-training mechanism that involves iterative slice relabeling/removal and SQA-Net retraining (green box in Fig. 1). Specifically, we first predict the quality of each slice using the pre-trained SQA-Net and then keep slices that meet one of the following conditions: 1) the predicted label is identical to the prediction in previous iteration; 2) the label is predicted with high-confidence, i.e., its predction maximal probability beyonds a threshold. In addition to removing slices that do not meet the above criteria, we also relabel the selected slices with high-confidence predicted labels. We then retrain SQA-Net for the next iteration until accuracy improvement is minimal.

### E. Volume Self-Training

VQA-Net is employed to predict the volumetric quality based on the slice features extracted by SQA-Net. Both labeled and unlabeled volumes are utilized to train VQA-Net, where the initial quality ratings of unlabeled volumes are determined based on the following rules: 1) “Pass” if more than 60 percent of the slices in the volume are labeled as “pass”; 2) “Fail” if more slices are labeled as “fail” than “pass” and “questionable”; 3) “Questionable” if otherwise. However, the initial volumetric quality determined by the empirical rules is inaccurate due to the indistinct bounds of each class for different subjects. To cope with inaccurate volumetric labels, similar to slice self-training, volume self-training involves iterative volume relabeling/removal, i.e., VQA-Net retraining (blue box in Fig. 1). Specifically, we retrain our VQA-Net with volumetric data where 1) the predicted labels are identical to the prediction in previous iteration; or 2) the labels are predicted with high-confidence, i.e., maximal probabilities beyond a threshold. We also replace the labels of the selected volumes with the predicted labels, and remove volumetric data that do not meet the above criteria from the training dataset. We then retrain VQA-Net and repeat the whole process until the accuracy improvement is minimal.

### F. Subject Assessment

To ensure that image quality is sufficient for subsequent processing [49], we assign quality label for each subject by using the following rules: 1) “Pass” if more than 80 percent of the volumes in a subject is labeled as “pass”; 2) “Fail” if more than 20 percent of the volumes in a subject is labeled as “fail”; 3) “Questionable” if otherwise, where the volumetric quality ratings are annotated by the trained VQA-Net.

### G. Loss Function

We also use *L*_2_ regularization for the Conv and DSConv layers to avoid overfitting. In addition, to deal with data imbalance, we use a multi-class balanced focal loss [50] with *L*_2_ regularization for both SQA- and VQA-Net, i.e.,

(3) $$L\left(p_{t}\right)=-\alpha_{t}{\left(1-p_{t}\right)}^{\kappa }\text{log}\left(p_{t}\right)+\frac{\lambda }{2n_{w}}\sum_{w}{\left\|W_{w}\right\|}_{2}^{2}$$

where *p*_*t*_ (*t* = 1, 2, 3) are the predicted probabilities for “pass”, “questionable”, and “fail”, respectively. *κ* ≥ 0 is a focusing parameter, **W**_*w*_ is the *w*-th weight matrices of SQA-/VQA-Net, *λ* = 0.01 is a tuning parameter for *L*_2_ regularization, and *n*_*w*_ is the number of weight matrices. Here, the class weights *α*_*t*_ = max(*N*_1_, *N*_2_, *N*_3_)/*N*_*t*_ are used for balancing the contributions of different classes in imbalanced datasets. *N*_*t*_ is the number of slices associated with the *t*-th class.

## IV. Experiments

### A. Dataset

The experiments were conducted using dMRI data of pediatric subjects from one month to six years of age. The diffusion MR images were acquired using Siemens Prisma MRI scanners using a 32-channel head coil [51] and echo planar imaging sequence with TR/TE = 2640/88.6 ms, voxel size 1.5mm×1.5mm×1.5mm, and *b* =0, 500, 1000, 1500, 2000, 2500, 3000 s/mm^2^ with a total of 144 non-collinear gradient directions. As shown in Table I, the images were separated into three datasets: 1) Training dataset; 2) Testing dataset; and 3) Unlabeled dataset.

**Fig. 3.**
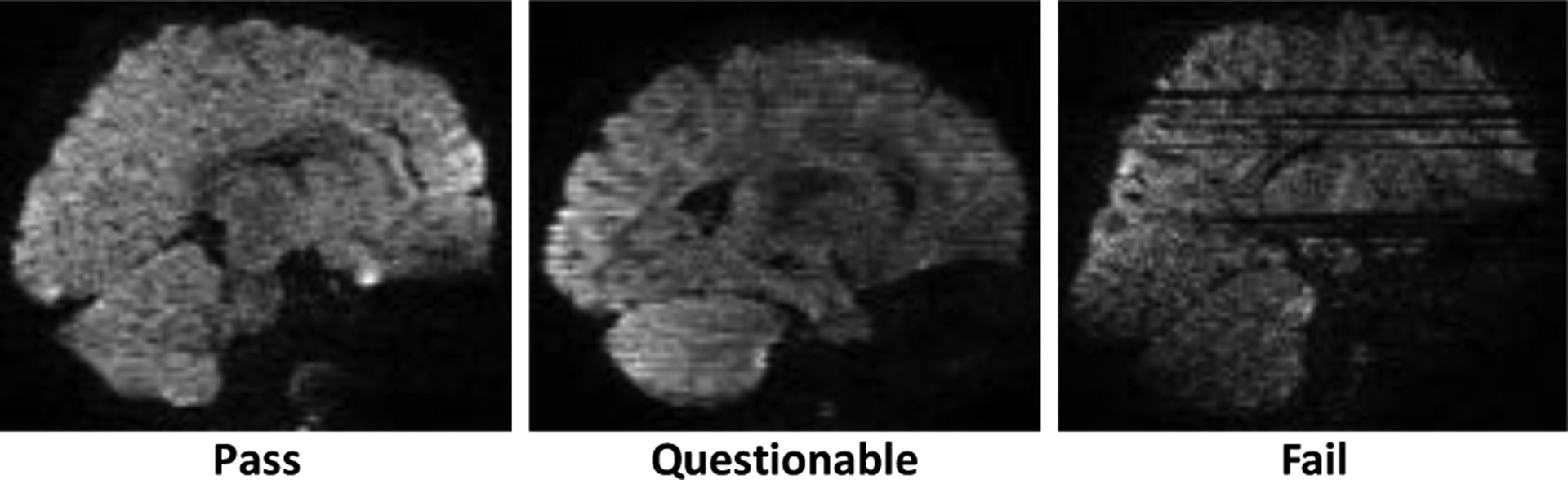
Example of image slices labeled as “pass” (no/minor artifacts), “questionable” (moderate artifacts), and “fail” (heavy artifacts).

A total of 151 volumes were acquired for each subject. 11778 unlabeled volumes and 3624 testing volumes were, respectively, extracted from 78 unlabeled subjects and 24 testing subjects. Since artifacts are more apparent when viewed using sagittal slices, 9000, 706680, and 217440 sagittal slices were extracted respectively from the 150 labeled volumes, 11778 unlabeled volumes, and 3624 testing volumes. Each slice was zero-padded to 144×144, min-max intensity normalized, and labeled according to the volume it belongs to. For the training data, subject-wise annotation was performed. For the testing data, more accurate image quality annotation was performed volume-wise. Fig. 3 shows examples of slices labeled as “pass” (no/minor artifacts), “questionable” (moderate artifacts), and “fail” (heavy artifacts).

### B. Implementation Details

The proposed method was implemented using Keras with Tensorflow backend. Evaluation was based on a machine with a CPU (i.e., Intel i7-8700K) and a GPU (i.e., NVIDIA GeForce GTX 1080Ti 11GB). The RMSprop optimizer was utilized for training with initial learning rate set to 1×10^−5^ and decay rate set to 5×10^−8^. To avoid overfitting, the data were augmented via rotation from 0 to 30 degrees and horizontal flipping. Semi-supervised learning, and slice and volume self-training were repeated twice with the same probability threshold described in Section IV–E.

### C. Evaluation Metrics

We evaluated our method by using three performance metrics, including classification accuracy (ACC), sensitivity (SEN), and specificity (SPE), which are defined as

(4) $$\text{ACC}=\frac{\text{TP}+\text{TN}}{\text{TP}+\text{TN}+\text{FP+FN}},\text{SEN}=\frac{\text{TP}}{\text{TP+FN}},\text{SPE}=\frac{\text{TN}}{\text{FP+TN}},$$

where TP, TN, FP and FN denote the numbers of true positives, true negatives, false positives, and false negatives, respectively. In addition, we also evaluated the computational efficiency of our method by using six metrics, i.e., the number of parameters (NoP), the maximal dimension (MD), and GPU and CPU time cost (TC) for slice-wise, volume-wise and subject-wise quality assessment.

### D. Compared Methods

To verify the effectiveness of the proposed IQA method, we compared it with several learning based methods:

- Supervised learning with fine tuning [20]: InceptionV3 [52], ResNet [53], VGG [54];
- Supervised learning with hand-crafted features: random forest with features extracted via Gabor filters;
- Unsupervised learning: *k*-means clustering [55].

For subject-wise IQA, our subject assessment strategy is applied to the volume-wise quality ratings given by these methods.

To verify the effectiveness and superiority of NLDR block and NLR block on constructing SQA-Net, we also compared with 3 ablated versions of SQA-Net:

- Depthwise separable residual (DSR) network : The NLDR block and NLR block are substituted with a DSR block with 128, 256, and 512 channels;
- DSR+NLR network: The NLDR block is substituted with a DSR block with 128 and 256 channels;
- NLDR+NLR network: The DSR block is substituted with a NLDR block with 32 and 64 channels.

Note that SQA-Net is a DSR+NLDR+NLR network. These comparison networks were trained in similar fashion as our proposed SQA-Net.

### E. Determination of Thresholds

The thresholds used for semi-supervised learning, and slice/volume relabeling and removal in slice/volume self-training are core factors that affect the overall performance. We determined the thresholds based on the following considerations.

The thresholds in slice/volume self-training (i.e., TH-S and TH-V, respectively) are utilized to relabel or remove slices/volumes with noisy labels, and to retain highly confident slices/volumes for network retraining. Similarly, the threshold in iterative semi-supervised learning is utilized to select confidently annotated pseudo-labeled slices, they share the same threshold parameter (i.e., TH-S). Besides, as these thresholds are used to determine highly confident slices/volumes, we set these threshold values according to the training accuracy. From our experiments, the slice and volume training accuracies are both greater than 0.85. Thus, we consider 3 possible threshold values for TH-S and TH-V, i.e., 0.7, 0.8 and 0.9.

**Fig. 4.**
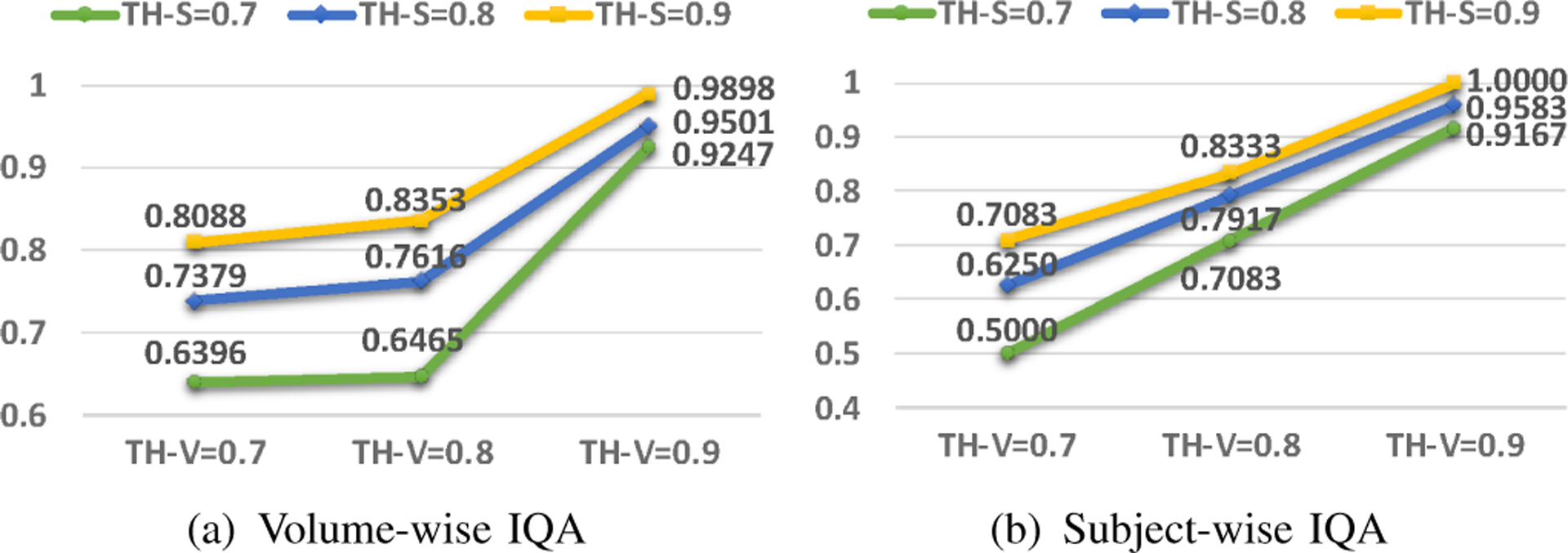
The accuracy of VQA and SQA with three different threshold values in slice and volume self-training. TH-S: threshold used in slice self-training, TH-V: threshold used in volume self-training.

Fig. 4 shows the comparison results of volume- and subject-wise IQA using different combinations of these three threshold values. From the figure, we can see that the performance of volume- and subject-wise IQA are improved whenever a larger TH-S or TH-V value is used, and the best performance is achieved when both thresholds are set to 0.9. Thus, in subsequent experiments, we set both TH-S and TH-V to 0.9. Note that increasing this threshold further (e.g., 0.99) will significantly reduce the amount of confident slices/volumes that can be used for further training to improve performance.

### F. Performance Comparison

**Fig. 5.**
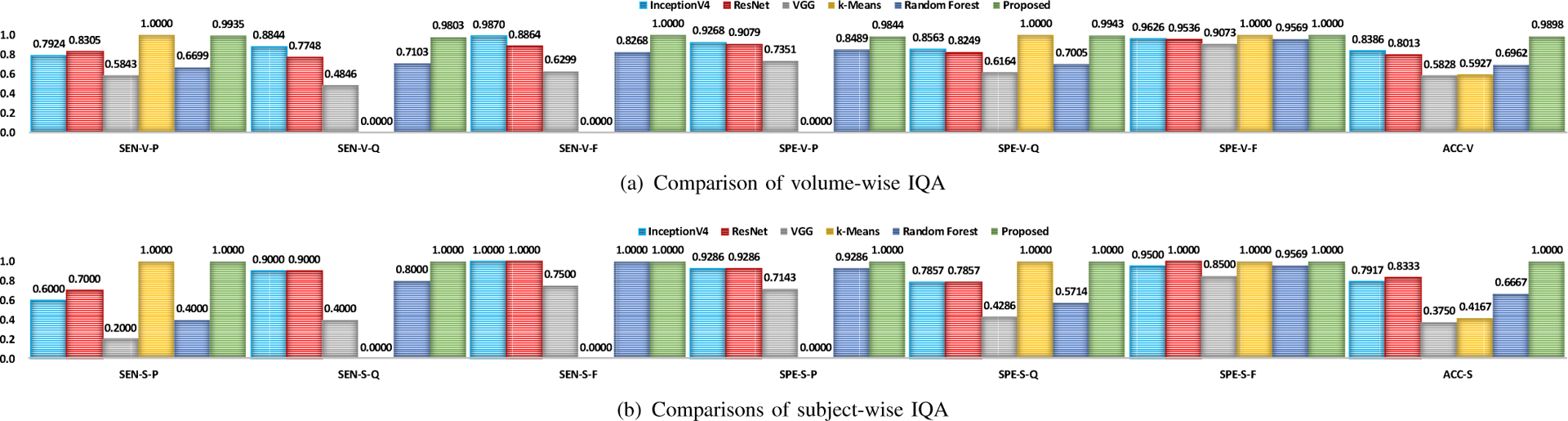
Comparison of volume- and subject-wise IQA. Evaluation metrics are denoted in the following form: metric (SEN/SPE/ACC) - stage (V/S) - quality (P/Q/F/-).

Fig. 5 shows the evaluation results in terms of sensitivity, specificity, and accuracy for volume- and subject-wise IQA of the testing dMRI data, using different IQA methods, i.e., InceptionV3 ResNet, VGG, *k*-means, random forest, and our method. From Fig. 5, our method significantly outperforms all compared methods for all evalution metrics except sensitivity to “pass” volumes (i.e., SEN-V-P). The highest performance for SEN-V-P is achieved by *k*-means. However, *k*-means is actually overfitting, as it yields zero sensitivity to “questionable” volumes (i.e., SEN-V-Q) and “fail” volumes (i.e., SEN-V-F). This might be caused by class imbalance in the training data.

### G. Ablation Study

#### 1) Computational Efficiency:

Computational efficiency was evaluated based on six metrics, i.e., number of parameters (NoP), maximal dimension (MD), GPU and CPU time cost (TC) of slice-, volume- and subject-wise IQA, as summarized in Table II.

For comparison, we summarized in Table II the TCs of manual assessment based on two experienced radiologists: slice, volume and subject TCs, respectively, are ~1s, ~22s, and ~3000s. These are much longer than automatic IQA.

Compared with DSR and DSR+NLR, the NLR block reduces the maximal dimension from 512 to 256 and reduces the NoP by over two times. This reduces the GPU and CPU slice, volume, and subject TCs by over 3.5%, and both the GPU slice and subject TCs by over 11.4%.

We can see that DSR+NLR with NLDR+NLR have the same MD, whereas the NoP of NLDR+NLR is over 50% larger than that of DSR+NLR due to the nonlocal network block integrated in the NLDR block. This causes the GPU and CPU slice, volume and subject TCs to be increased by at least a factor of 2.

NLDR+NLR and DSR+NLDR+NLR have the same maximal dimension and similar NoPs. The GPU and CPU slice, volume and subject TCs are reduced by over 26%, particularly the CPU slice and volume TCs, which are reduced by over almost four times due to the substitution of the first two NLDR blocks with DSR blocks.

The analysis above shows that the DSR and NLR blocks improve the computational efficiency of the overall network. Although our method is not the most computationally efficient, the GPU and CPU TCs differ only slightly than DSR and DSR+NLR. The proposed method is hence suitable for fast dMRI IQA.

#### 2) Network Efficiency:

Tables III and IV show respectively the confusion matrix, sensitivity, specificity, and accuracy of the compared methods for volume- and subject-wise IQA of the testing dMRI data. As shown in Table IV, the proposed method (DSR+NLDR+NLR network) yields the best volume- and subject-wise IQA performance when compared with other methods in terms of sensitivity, specificity, and accuracy.

Compared with DSR and DSR+NLR, the NLR block improves the volume sensitivity for “pass” and “questionable” by over 10.2%, the volume specificity for all quality ratings by over 4.5%, and the accuracy of volume- and subject-wise IQA by over 16.3%. Compared with DSR+NLR and NLDR+NLR, replacing DSRs with NLDRs greatly improves sensitivity, specificity, and accuracy in both volume- and subject-wise IQA, e.g., over 24.0% improvements in sensitivity to “pass” and “fail” volumes, specificity to “questionable” volumes and subjects, and accuracy of volume- and subject-wise IQA. Compared with NLDR+NLR and DSR+NLDR+NLR (i.e., the proposed method), where the DSR block instead of the NLDR block is used to extract low-level features, the performance of volume- and subject-wise IQA is further improved.

It follows from the observations above that DSR, NLDR and NLR blocks enhance the performance of the overall network and make it more suitable for hierarchical IQA.

### H. Discordant Case Analysis

**Fig. 6.**
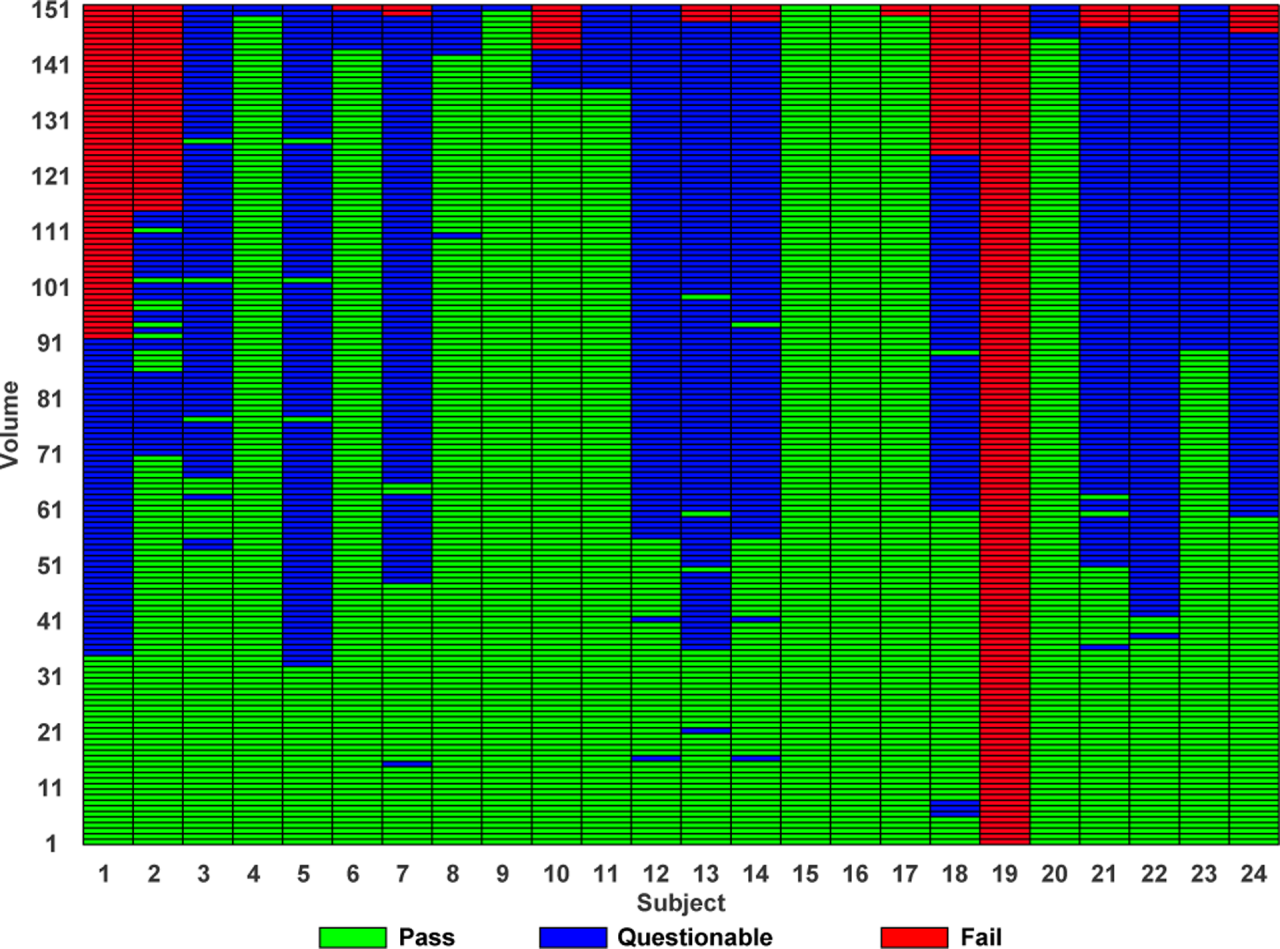
Volume- and subject-wise QA using our method. All volumes are sorted in the sequence of “fail”, “questionable” and “pass” from top to bottom.

Fig. 6 shows the predicted quality rating for each volume of the testing subjects using our volume-wise IQA method. From Table III(d), Table IV(d), and Fig. 6, we can observe that the actual quality ratings disagree with our predictions only for “pass” and “questionable” volumes. That is, our method may occasionally mistaken “pass” and “questionable” volumes.

**Fig. 7.**
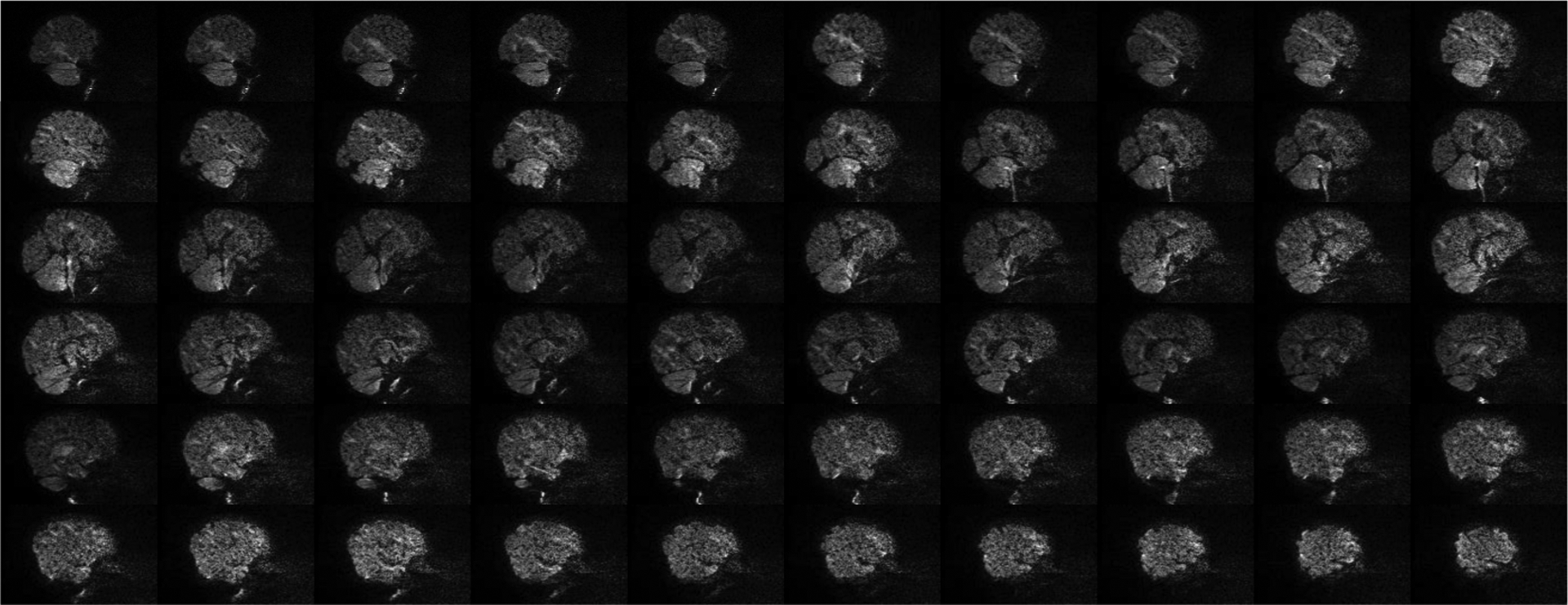
Slices belonging to a discordant “pass” volume, which is mis-predicted as “questionable”. (*b* = 2000 s/mm^2^)

**Fig. 8.**
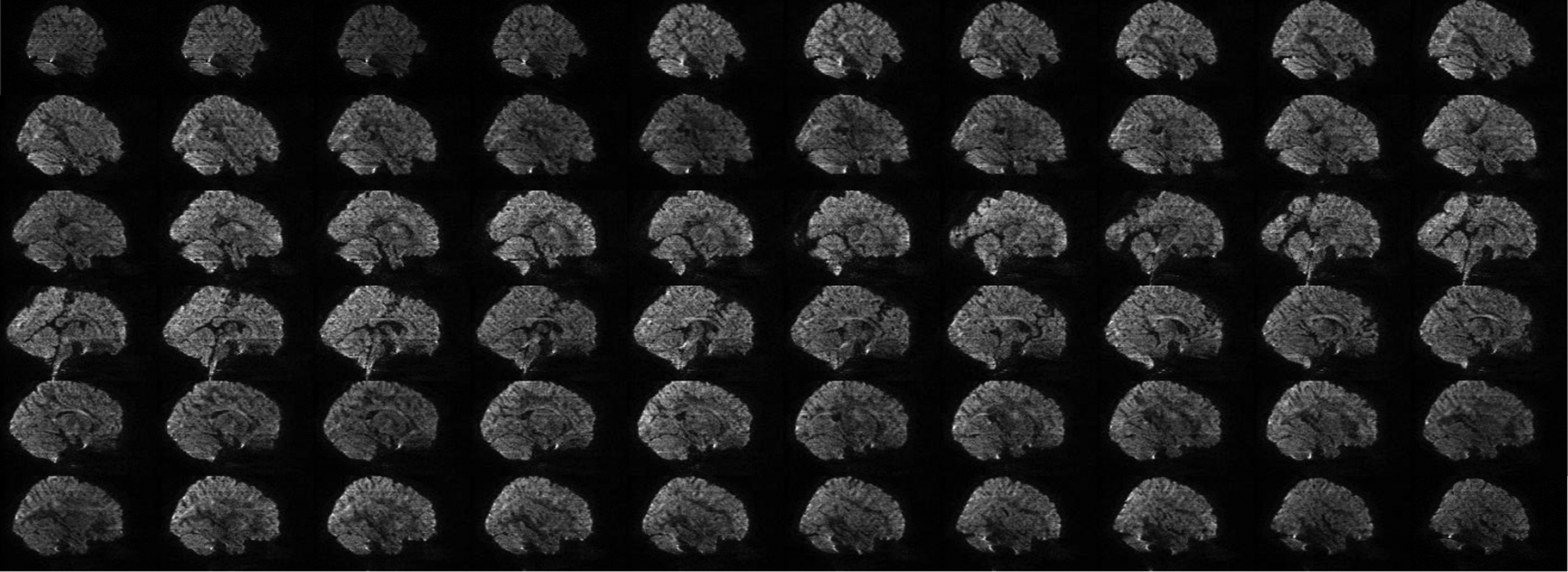
Slices belonging to a discordant “questionable” volume, which is mis-predicted as “pass”. (*b* = 1500 s/mm^2^)

Inspecting retrospectively all “pass” and “questionable” training volumes, subtle degradation such as contrast reduction and local fuzziness, as shown in Figs. 7 and 8, can confuse the IQA network and cause mispredictions.

### I. Effectiveness of Semi-Supervised Learning

We evaluated the effectiveness of semi-supervised learning by comparing SQA-Net with and without semi-supervised learning. The subsequent volume assessment was implemented using VQA-Net without volume self-training.

**Fig. 9.**
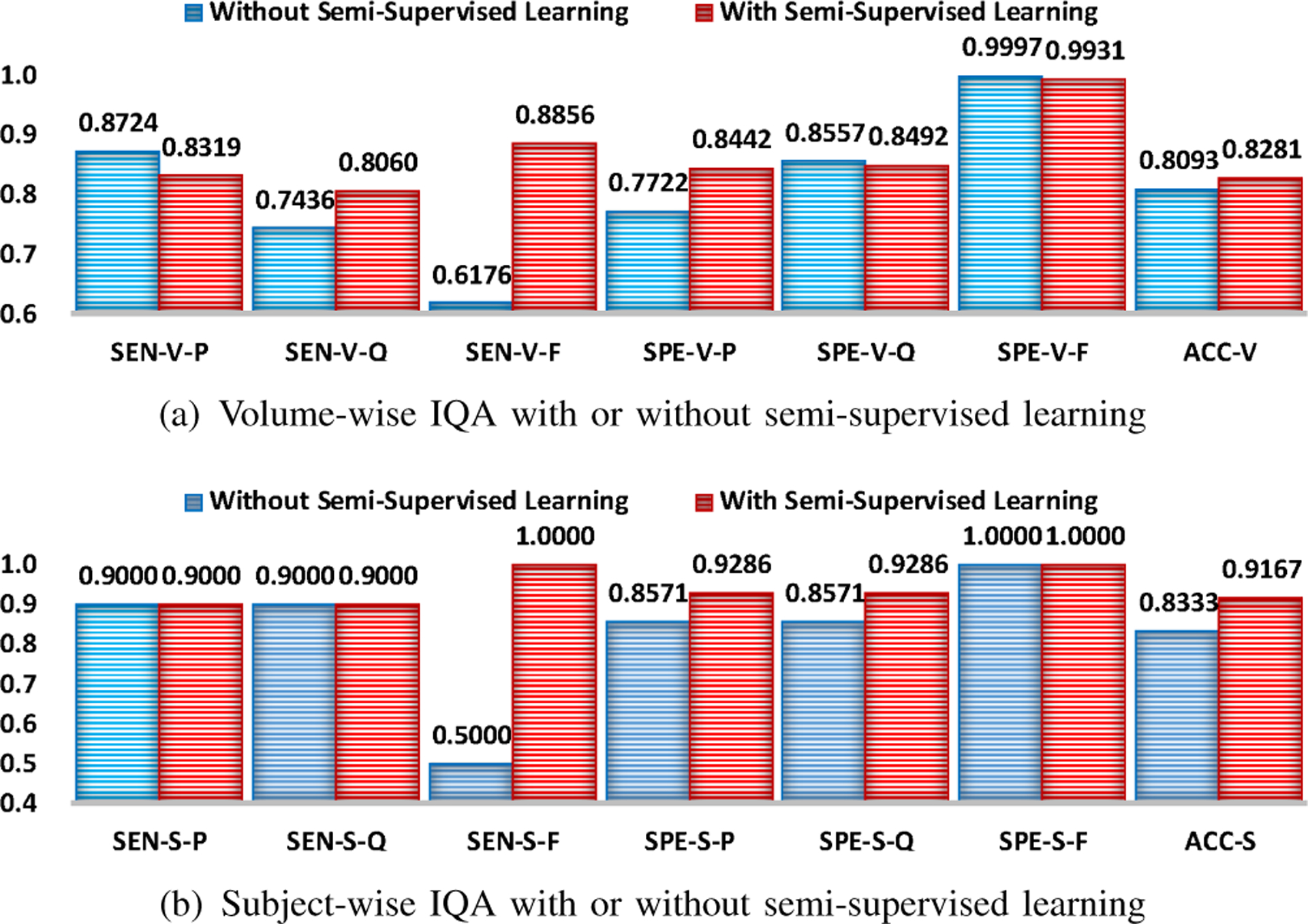
Volume- and subject-wise IQA using our method trained with and without semi-supervised learning. Self-training is removed for both cases in this experiment. Evaluation metrics are denoted in the following form: metric (SEN/SPE/ACC)-stage (V/S)-quality (P/Q/F/-).

From Fig. 9, we can observe that semi-supervised learning improves volume- and subject-wise IQA performance. The greatest improvement is the sensitivity to “fail” volumes or subjects. Although there is performance drop for some performance metrics (e.g., sensitivity to “pass” volumes, and specificity to “questionable” and “fail” volumes), the degree of degradation is minimal. Thus, in overall, the pseudo-labeled slices that were selected via semi-supervised learning to retrain SQA-Net are beneficial for improving the performance of volume- and subject-wise IQA.

### J. Effectiveness of Slice Self-Training

We evaluated the effectiveness of slice self-training by comparing the performance of SQA-Net with and without slice self-training. In the latter, slice self-training is removed from the training of SQA-Net. The subsequent volume assessment was implemented using VQA-Net without volume self-training.

**Fig. 10.**
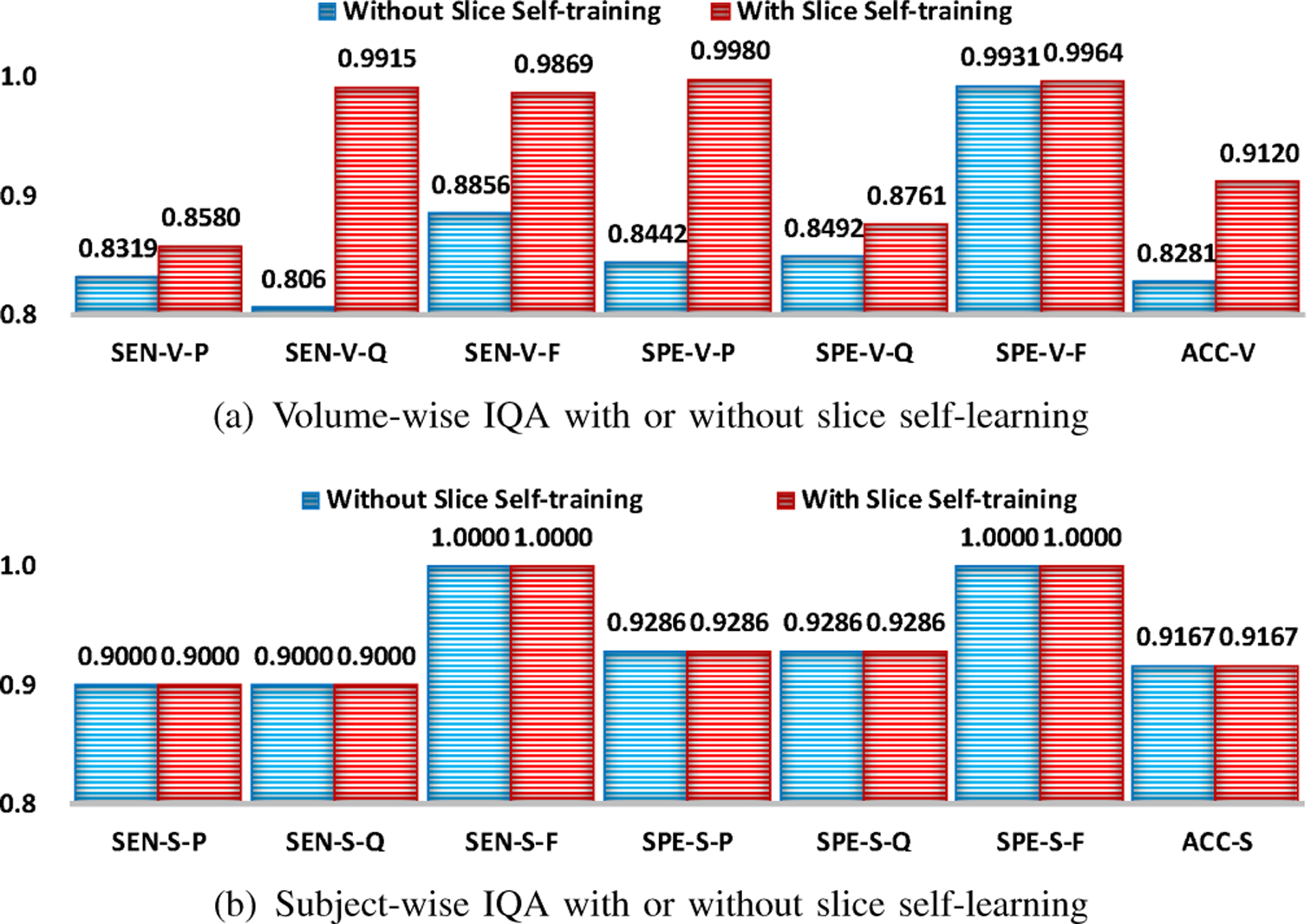
Volume- and subject-wise IQA using our method trained with and without slice self-training. Evaluation metrics are denoted in the following form: metric (SEN/SPE/ACC)-stage (V/S)-quality (P/Q/F/-).

From Fig. 10, we can observe that slice self-training improves the volume- and subject-wise IQA performance, especially sensitivity to “questionable” and “fail” volumes, specificity to “pass” volumes, and accuracy of volume-wise IQA. This indicates that retaining confident slices and relabeling/removing noisy slices via the self-training strategy improve the performance of subsequent volume- and subject-wise IQA.

### K. Effectiveness of Volume Self-Training

We evaluated the effectiveness of the volume self-training by comparing the performance of VQA-Net with and without volume self-training.

**Fig. 11.**
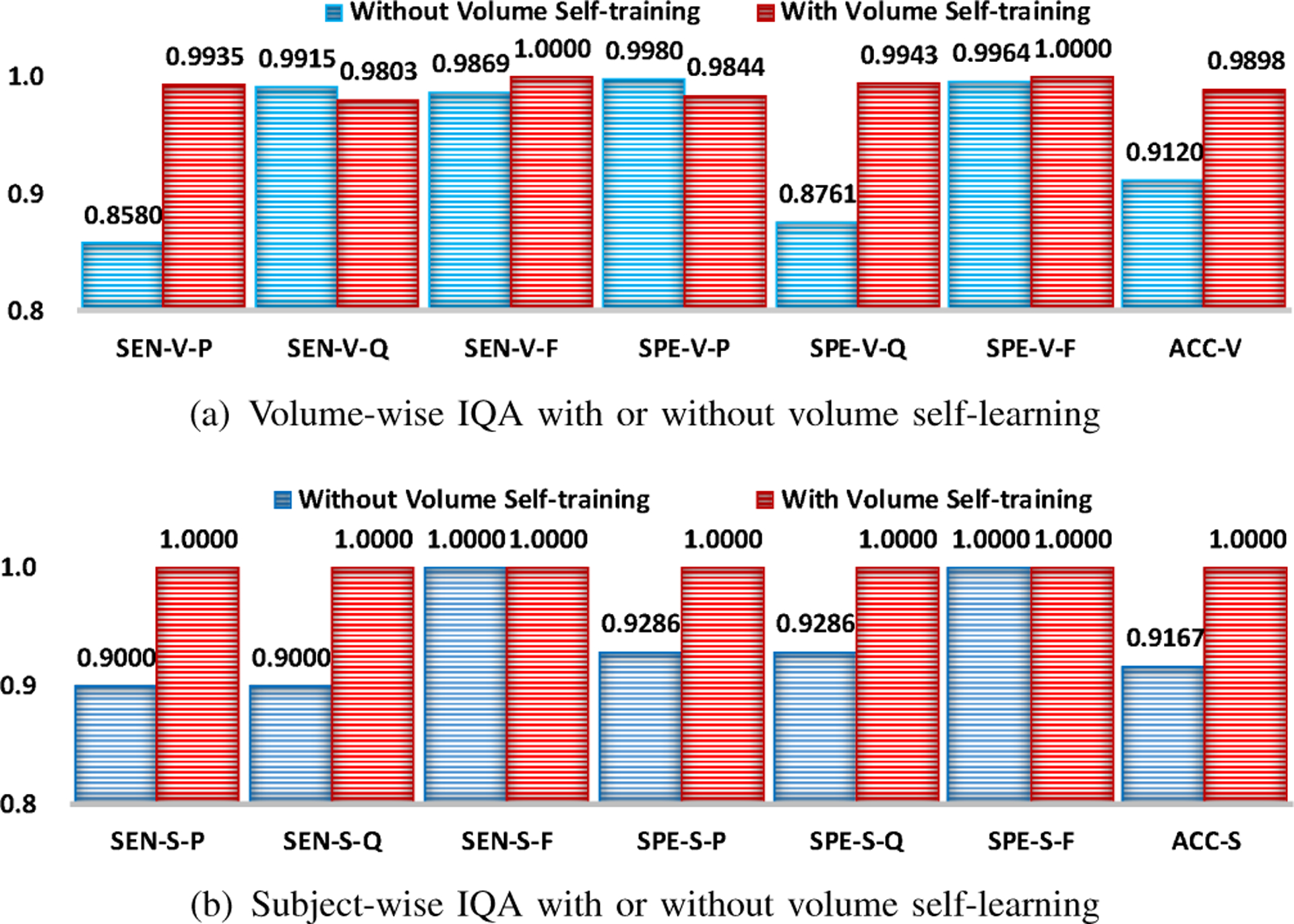
Volume- and subject-wise IQA using our model that was trained with and without volume self-training. SQA-Net was pre-trained via semi-supervised learning and subsequent VQA-Net did not use volume self-training. Evaluation metrics are denoted in the following form: metric (SEN/SPE/ACC)-stage (V/S)-quality (P/Q/F/-).

From Fig. 11, we can observe that volume self-training improves volume- and subject-wise IQA performance, especially sensitivity to “pass” volumes, specificity to “questionable” volumes and accuracy of volume-wise IQA. This indicates that retaining volumes with reliable labels and relabeling volumes via self-training strategy can improve the performance of subsequent subject-wise IQA. Although there is performance drop for some performance metrics (e.g., sensitivity to “questionable” volumes and specificity to “pass” volumes, the degree of degradation is minimal.

## V. Discussion

### A. Comparison With Previous Work

In general, no-reference image quality assessment (NR-IQA) methods can be divided into metric-based methods and learning-based methods. Metric-based methods aim to devise a metric to measure severity of an artifact, and thus the quality of an image. The major drawback of metric-based methods is that they are often artifact-specific, i.e., they often use different metrics to measure different artifacts. For example, the image blurriness can be assessed based on image sharpness via edge analysis [56–59], domain transform [60, 61], or pixel-wise statistics [62, 63]. On the other hand, the image noisiness can be estimated by measuring the noise variance via wavelet transform [64], block variance estimation [65–67] and filtering [68–70]. Currently, most metric-based methods focus on measuring blurriness and noisiness of an image, which might not necessarily cover all types of artifacts in diffusion imaging. In contrast, learning-based methods, such as SVM based [17, 71–73] and deep learning based [20, 74–79], aim to replicate the assessments of human raters using labeled (and unlabeled) data. The main difference between SVM-based and deep-learning based methods is that the former relies on hand-crafted features, whereas the latter can automatically extract features that are pertinent to IQA.

For deep learning-based methods, we discuss the differences between our method and previous deep learning based methods in two aspects, i.e., hierarchical organization and learning algorithm.

#### 1) Hierarchical Organization:

Compared with the existing learning-based IQA methods that assess 2D [74–76] and 3D anatomical [77, 78], or 4D functional [20, 79] neuroimages, our method adopts 2D slices as inputs to develop a hierarchical IQA model. Specifically, we hierarchically construct multi-scale (i.e., slice-, volume-, and subject-wise) quality assessment networks, where the outputs of the preceding networks are used as inputs to subsequent network. Furthermore, our hierarchical method can be adjusted flexibly according to data availability, i.e., when a large labeled dataset is available, the semi-supervised learning process can be omitted; when a small labeled dataset and a large unlabeled dataset are available, the whole proposed framework can be employed.

#### 2) Learning Algorithm:

The learning algorithm of our method differs from that of existing learning-based IQA methods [74–77, 79]. In contrast to existing learning-based methods that require sufficient labeled data for training, our method only require a small number of labeled training data, which are progressively expanded by iteratively including confident slices into the training dataset using semi-supervised learning. Moreover, unlike most previous methods that assume “clean” labels, our method considers multi-scale (i.e., slice/volume) noisy labels during network training, and iteratively retains high-confident data and relabel noisy data via our self-training strategy. In addition, the hierarchical training mechanism of our method also implies that less training data are required to optimize network parameters, compared with end-to-end networks.

### B. Limitations and Future Work

While our method achieved near-perfect performance for dMRI IQA, its generalizabillity can be further improved in the future by addressing the following limitations and challenges.

In our current implementation, the performance refinement strategies, i.e., semi-supervised learning and slice/volume self-training, were carried out individually for time efficiency. Alternatively, these refinement processes can be trained end-to-end.

Furthermore, currently whole 2D slices are used for training, ignoring the fact that the background region may have little information about motion artifacts. To focus more on the brain, a brain detection model can be incorporated into our framework to determine a brain mask as additional input to the SQA-Net.

## VI. Conclusion

In this paper, we have proposed a hierarchical deep learning method for QA of pediatric diffusion MR images. Our framework performs IQA slice-, volume-, and subject-wise. Our method requires only a small amount of quality-annotated images for pretraining the SQA-Net, which is then used to pseudo-label unannotated image slices to expand the training dataset with confidently pseudo-labeled images. Our IQA network is then further refined via subsequent slice and volume self-training. Our method can cope with label noise effectively, affording tolerance to inevitable rater errors, minimizing the requirement of labeled data and reducing the time of manual labeling. Experimental results verify that the proposed method yields near perfect IQA accuracy at a low computational cost.

## Figures and Tables

**TABLE I:** Datasets used for evaluation

Training Dataset (Unit: Volume)			Unlabeled Dataset (Unit: Volume)	Testing Dataset (Unit: Volume)		
Pass	Ques	Fail		Pass	Ques	Fail
85	25	40	11778	2148	1170	306

**TABLE II:** Computation efficiency of different methods

Method	NoP		MD	Slice TC		Volume TC		Subject TC	
	SQA-Net	VQA-Net		GPU	CPU	GPU	CPU	GPU	CPU
DSR	0.74M	2.11M	512	0.149s	0.152s	7.652s	8.611s	16.773s	258.679s
DSR+NLR	0.32M	2.11M	256	0.132s	0.151s	7.141s	8.310s	14.421s	237.999s
NLDR+NLR	0.50M	2.11M	256	0.275s	0.834s	15.903s	45.692s	35.312s	571.352s
DSR+NLDR+NLR (Proposed)	0.49M	2.11M	256	0.202s	0.210s	9.321s	11.635s	18.786s	263.062s
Manual Assessment	-		-	~ls		~22s		~3000s	

**TABLE III:** Confusion matrices

(a) DSR							
Image Quality		Predicted					
		Pass		Ques		Fail	
		Volume	Subject	Volume	Subject	Volume	Subject
Actual	Pass	1254	4	892	6	2	0
	Ques	132	1	1000	9	38	0
	Fail	10	0	62	1	234	3
(b) DSR+NLR							
Image Quality		Predicted					
		Pass		Ques		Fail	
		Volume	Subject	Volume	Subject	Volume	Subject
Actual	Pass	1569	7	579	3	0	0
	Ques	63	1	1102	9	5	0
	Fail	18	0	66	1	222	3
(c) NLDR+NLR							
Image Quality		Predicted					
		Pass		Ques		Fail	
		Volume	Subject	Volume	Subject	Volume	Subject
Actual	Pass	1952	9	196	1	0	0
	Ques	33	0	1135	10	2	0
	Fail	0	0	15	0	291	4
(d) DSR+NLDR+NLR							
Image Quality		Predicted					
		Pass		Ques		Fail	
		Volume	Subject	Volume	Subject	Volume	Subject
Actual	Pass	2134	10	14	0	0	0
	Ques	23	0	1147	10	0	0
	Fail	0	0	0	0	306	4

**TABLE IV:** Sensitivity, specificity, and accuracy

(a) DSK						
Image Quality	SEN		SPE		ACC	
	Volume	Subject	Volume	Subject	Volume	Subject
Pass	0.5838	0.4000	0.9038	0.9286	0.6865	0.6667
Ques	0.8547	0.7000	0.6112	0.5000		
Fail	0.7647	0.7500	0.9879	1.0000		
(b) DSR+NLR						
Image Quality	SEN		SPE		ACC	
	Volume	Subject	Volume	Subject	Volume	Subject
Pass	0.7304	0.7000	0.9451	0.9286	0.7983	0.7917
Ques	0.9419	0.9000	0.7372	0.7143		
Fail	0.7255	0.7500	0.9985	1.0000		
(c) NLDR+NLR						
Image Quality	SEN		SPE		ACC	
	Volume	Subject	Volume	Subject	Volume	Subject
Pass	0.9088	0.9000	0.9776	1.0000	0.9321	0.9583
Ques	0.9701	1.0000	0.9140	0.9286		
Fail	0.9510	1.0000	0.9994	1.0000		
(d) DSR+NLDR+NLR						
Image Quality	SEN		SPE		ACC	
	Volume	Subject	Volume	Subject	Volume	Subject
Pass	0.9935	1.0000	0.9844	1.0000	0.9898	1.0000
Ques	0.9803	1.0000	0.9943	1.0000		
Fail	1.0000	1.0000	1.0000	1.0000		
